# Forecasting stock prices using long short-term memory involving attention approach: An application of stock exchange industry

**DOI:** 10.1371/journal.pone.0319679

**Published:** 2025-03-18

**Authors:** Muhammad Idrees, Maqbool Hussain Sial, Najam Ul Hassan

**Affiliations:** 1 Department of Economics and Quantitative Methods, Dr. Hasan Murad School of Management (HSM), University of Management and Technology (UMT), Lahore, Pakistan; 2 Department of Economics, Thal University, Bhakkar, Pakistan; Khalifa University, UNITED ARAB EMIRATES

## Abstract

The Stability of the economy is always a great challenge across the world, especially in under developed countries. Many researchers have contributed to forecasting the Stock Market and controlling the situation to ensure economic stability over the past several decades. For this purpose, many researchers have built various models and gained benefits. This journey continues to date and will persist for the betterment of the stock market. This study is also a part of this journey, where four learning-based models are tailored for stock price prediction. Daily business data from the Karachi Stock Exchange (100 Index), covering from February 22, 2008 to February 23, 2021, is used for training and testing these models. This paper presenting four deep learning models with different architectures, namely the Artificial Neural Network model, the Recurrent Neural Network with Attention model, the Long Short-Term Memory Network with Attention model, and the Gated Recurrent Unit with Attention model. The Long Short-Term Memory with attention model was found to be the top-performing technique for accurately predicting stock exchange prices. During the Training, Validation and Testing Sessions, we observed the R-Squared values of the proposed model to be 0.9996, 0.9980 and 0.9921, respectively, making it the best-performing model among those mentioned above.

## Introduction

Karachi, Lahore and Islamabad Stock Exchanges were merged and renamed as Pakistan Stock Exchange (PSX) on 11^th^ January 2016. In May 2017, it became a component of the MSCI Emerging Market Index. Currently, PSX is associated with 400 brokerage houses and 21 asset management companies. [1]. On the basis of non-linearity, non-parametricity, and deterministic chaos, the stock market is considered a complex system. This complexity arises from changes in stock availability, the fluid nature of money, speculative behavior, stock market news, human behavior, and fluctuations in international currency exchange rates. [2]. In recent years, forecasting stock prices has become a critical focus in research. Initially, researchers tried to develop a linear relationship between macroeconomic variables and the stock market. However, when analyzing stock market index returns, the identification of nonlinear trends remains challenging, [2] initiated a significant shift in researchers’ focus on nonlinear prediction methods for stock returns. Despite the extensive development of literature on nonlinear modeling of stock prices, researchers must specify a nonlinear model before estimation. Stock Market returns have a great noise, non-linearity and uncertainty, therefore defining a nonlinear model in advance causes many challenges. [3]. Previous studies reflect different methods for predicting stock returns using different input variables. Many studies use input data from a single time series, whereas others incorporate broader market insights and macroeconomic factors. [3].

Machine learning plays an important role in discovering hidden patterns and features during the learning process. It helps in predicting complex input-output relationships, demonstrating its effectiveness in determining optimal pricing strategies for organizations. [3]. The research of stock prices is especially effective in deep learning, using different techniques and methods. USA, Canada, UK, Hong Kong and Japan adopted this technique. They use machine learning to train and adeptly retrieve stock data. [3,4]. [2] used Support Vector Regression (SVR) approach for input selection with different windowing operators as part of data pre-processing steps for predicting stock exchange prices. [4] predicting Karachi Stock Exchange (KSE 100) index closing using Artificial Neural Networks (ANN), Autoregressive Moving Averages (ARMA) modeling with news sentiments. [5] employed Artificial Neural Networks (ANN) as a machine learning method, adding different windowing operators for input selection within the steps of data pre-processing. The attention model has gained significant success across different fields, including environmental studies [6], sociology [7,8], finance [9,10] engineering [11,12] and emotion detection [13].

### Research gap

The stock market is a dynamic and complex system shaped by several factors such as human behavior, market news, and diverse economic conditions. Although, Long-Short Term Models (LSTM) have the capability to capture dependencies in sequential data, they often face challenges in focusing on the relevant segments of that data. To address these challenges, attention mechanisms are integrated into LSTM models, enhancing their ability to focus on key patterns within time series data, leading to more accurate insights.

### Main contributions

In this research, we present a new architecture to predict stock prices. In this approach, we integrate LSTM networks with an attention mechanism to identify and capture the most relevant long-term dependencies in historical stock data. Additionally, we evaluated the LSTM-Attention model using SHAP, a framework for AI model interpretability. Furthermore, its performance was compared against other sequential data models.

### Related work

Forecasting stock prices has been a key area of financial research, with early models relying on linear techniques such as Autoregressive Moving Averages (ARMA) and other methods like Support Vector Machines (SVM), which can deal both linear and non-linear relationships, to predict prices [14]. However, these models face challenges in capturing the stock market’s inherent nonlinearity and volatility. [15]. The adoption of machine learning led to significant improvements, with Artificial Neural Networks (ANNs) being applied to stock prediction in various markets, including the Karachi Stock Exchange (KSE). [16]. Recent advancements in deep learning, particularly with Long Short-Term Memory (LSTM) networks, have further enhanced accuracy by modeling complex temporal patterns. [17]. Combining LSTM with attention mechanisms improves performance by focusing on essential time steps, and outperforming traditional models in financial applications. [18]. This approach has been successfully applied in different markets, such as the U.S. market and major Asian exchanges, demonstrating its robustness. [19]. Building on this foundation, the present study compares the performance of LSTM with attention to that of ANN, RNN-attention, and GRU-attention for predicting KSE prices, showing superior accuracy during both training and testing phases.

This research study focuses on forecasting the closing price of the Karachi Stock Exchange (KSE) 100 index using LSTM-attention modeling. This study consists of three sections. The first section provides information on data collection, LSTM, the drawbacks of LSTM, and the reasons for using LSTM-attention. The Second section will cover the proposed model architecture briefly. Finally, the third section will present the results, discussions, and conclusions.

## Materials and methods

### Data collection and availability

The data for this research have been downloaded from https://www.kaggle.com/datasets/zusmani/pakistan-stock-exchange-kse-100, which contains information about the KSE-100 index (Karachi Stock Exchange). The dataset consists of seven features of the KSE-100 index, which are as follows:

- Date → Current day when stock prices understudied.
- Open → First price of the stock at the time of market open.
- High → Highest price reached during the session.
- Low → Lowest price reached during the session.
- Close → Last price of the stock at the time of market close.
- Change  → Difference between current day close price and previous day close price.
- Volume  → Total number of shares trade during the day.

The Data has 3221 rows. It spans the period from February 22, 2008, to February 23, 2021. The data contain one entry per business day.

### Data pre-processing

Since there is a lot of variations in the data columns, i.e., the Open, High, Low, and Close Columns are in thousands, the Change Column is in tens or hundreds, and the Volume Column is in millions. To address this variation, the Standardization technique was adopted.


$$z=\frac{x-\mu }{s}$$ (1)

Where μands are the mean and standard deviation respectively.

In the training set, each feature is centered and scaled independently by calculating the relevant statistics on the samples. Moreover, for training purposes, the **Date** column has been dropped. Since future predictions are based on the past 10 days’ data, the original data (3221, 6) has been transformed to (3209, 10, 6). Finally, the data has been split into two portions: training and testing. The training data consist of 3009 samples, ranging from February 22, 2008, to May 6, 2020, while the testing data consist of 200 samples, from May 7, 2020, to February 23, 2021.

### Long short-term memory (LSTM)

**Fig 1:**
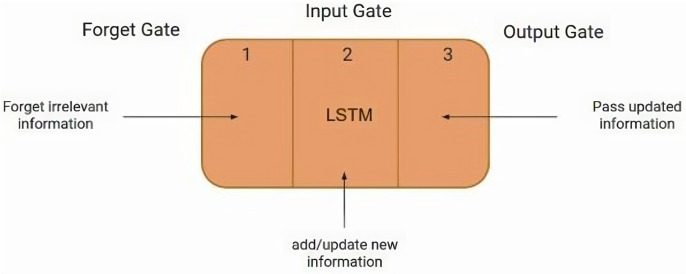
The basic structure of LSTM.

Introduced in 1997, the LSTM network has a specific structure that makes it suitable for processing and forecasting important events in time series data over extended intervals. [20]. The structure of LSTM is similar to that of a gated recurrent neural network (RNN), and uses a gating technique to manage the flow of information within the neural network. This mechanism builds upon the principles of a basic recurrent neural network. [21–23]. Within an LSTM, three control units, known as gates (input gate, output gate, and forget gate), play a pivotal role, as shown in Fig 1.

Among these, the input and forget gates are critical components that enable LSTM to maintain long-term dependencies. The input gate determines how much information from the current network state should be stored in the internal state. The forget gate determines which information from the past should be discarded. Lastly, the output gate controls how much information from the internal state should be transmitted to the external state at the current time.

### Attention

The attention mechanism plays a vital role in enhancing the performance of deep learning models, particularly in tasks involving sequential data such as stock price prediction, natural language processing, and time series forecasting. It was developed to address the limitations of traditional models such as Recurrent Neural Networks (RNNs) and Long Short-Term Memory (LSTM) networks. Instead of treating all input data equally, the attention mechanism enables the model to focus selectively on the most relevant parts of the input. In LSTM models, which are designed to capture temporal patterns in sequential data, the attention mechanism enhances the model’s ability to highlight key features at each time step, ensuring that significant inputs are prioritized while the impact of less important ones is minimized. [18]. This targeted focus is especially useful for long sequences, where crucial information might otherwise be lost among irrelevant data, potentially leading to challenges such as vanishing or exploding gradients. [24].

### LSTM attention

LSTM efficiently addresses gradient explosion issues. Overfitting may lead to poor performance on the test set during the training phase, which can be a significant issue. A dropout layer is added to improve the design of LSTM. Dimension Explosion problems arise in large-dimensional neural networks in LSTM because this model typically sets the input vector to a fixed, low dimension to achieve better results. To concentrate more effectively on influential parameters, this study introduces the attention mechanism. This method involves reserving the intermediate output of the LSTM encoder for the input sequence and training a model to selectively learn from these inputs, linking them to the output sequence when the model generates outputs. [25,26].

### Mean absolute error (MAE)

Mean Absolute Error (MAE) is defined as the mean of the absolute differences between predicted values and actual values.


$$MAE=\frac{1}{n}\overset{n}{\underset{i=1}{\sum }}\left|P-A\right|$$ (2)

Here P,Aandn denote the predicted value, actual value, and the total number of days respectively, in the data set. MAE precisely calculates the absolute deviation between the predicted actual values. MAE reflects the overall extent of forecasting errors without considering their directionality. The objective is to consistently minimize MAE in forecasting. MAE can be influenced by information transformations and the dimensions of the measurements. Notably, extreme forecast errors are nonexistent in MAE. [27].

### Mean average percentage error (MAPE)

Performance assessment is a critical element in research studies, enabling researchers to efficiently evaluate their findings. Different techniques have been used to measure the performance of experimental designs across different studies. In this study, MAPE is used for this purpose. MAPE is particularly useful for clearly illustrating the discrepancy between actual and predicted values. As a result, many researchers in financial analysis prefer using MAPE to assess the performance of their models. [28–30].

MAPE is also known as Mean Absolute Percentage Deviation (MAPD).

It is used to measure the precision of a fitted time series model, particularly in a trend estimation within statistics. The following MAPE formula is represented as a percentage:


$$MAPE=\left(\frac{1}{n}\left(\overset{n}{\underset{i=1}{\sum }}\frac{\left|A-P\right|}{A}\right)\right)\times 100$$ (3)

Here P,Aandn denote the predicted share prices, the actual share prices and the number of days, respectively, The result will be displayed as a percentage.

### Root mean squared error (RMSE)

RMSE is an important metric for evaluating the performance of a regression model. It calculates the mean difference between the predicted values from the model and the actual values, providing insight into the model’s accuracy in estimating the target value. A lower RMSE value indicates a more accurate model. Mathematically:


$$RMSE=\sqrt{\frac{1}{n}\left(\overset{n}{\underset{i=1}{\sum }}{\left(A-P\right)}^{2}\right)}$$ (4)

Here P,Aandn denote the predicted share prices, the actual share prices and the number of days, respectively. [27] suggests that RMSE is a reliable measure for evaluating overall forecast error.

### Model architecture

**Fig 2:**
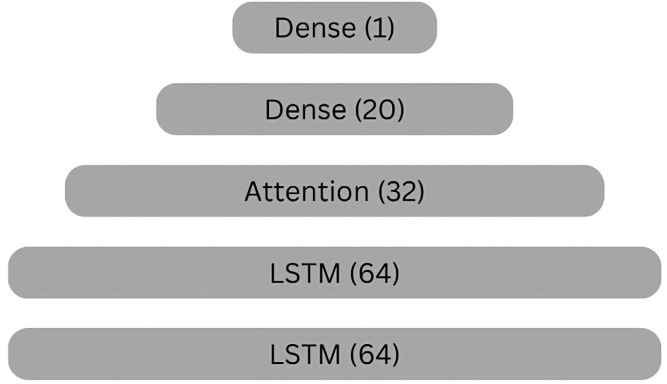
Abstract view of proposed model.

**Fig 3:**
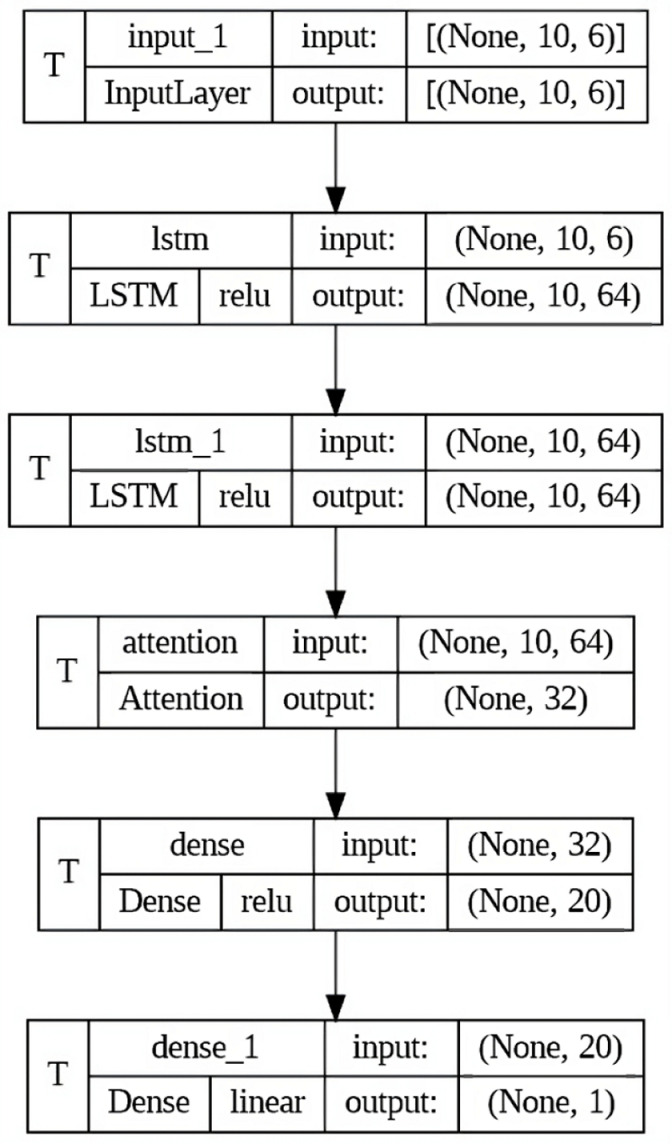
Detailed view/architecture of proposed model.

The neural network architecture is developed for sequence prediction tasks, presenting a formation of specialized layers designed to process sequential data efficiently, as shown in Figs 2 and 3. The proposed model is developed within the TensorFlow-Keras framework, facilitating ease of implementation and integration with existing deep learning frameworks.

**Input Layer:**

- The input layer determines the initial stage of data consumption and describes the shape of the input data tensor.
- The shape: (10, 6) indicates sequences of length 10, with each sequence containing vectors of size 6.
- This layer serves as the entry point for sequential data to be processed by the subsequent layers.

**LSTM Layers:**

- LSTM layers are important modules for modeling sequential data due to their capability to express temporal dependencies.
- Two LSTM layers are stacked sequentially, each arranged to output sequences (return_ sequences=True) rather than single outputs.
- Units: 64: Each LSTM layer consists of 64 memory units, granting the model’s capacity to train composite patterns within sequences.

**Activation function:**

- The Rectified Linear Unit (ReLU) activation function establishes non-linearity, enabling richer representations of temporal data.

**Attention layer:**

- The attention technique is integrated into the architecture to improve the model’s capability to emphasize relevant sections of input sequences.
- The attention layer is a convention operation designed to enhance the LSTM-based feature extraction process.
- Units: 32: Specifies the dimensionality of the attention space, allowing selective attention to salient features within the input sequences.

**Dense layers:**

- Following the attention technique, densely connected layers are engaged to further process the extracted features and produce predictive outputs.
- Two dense layers are included, each consisting to the hierarchical transformation of the input data.
- The first dense layer consists of 20 units with ReLU activation.
- The final dense layer contains a single unit, serving as the output layer for regression tasks.

**Optimizer:**

- Adam (Adaptive Moment Estimation) optimizer is commonly used for improving neural network models.
- Adam transforms the learning rate for each parameter, typically resulting in faster convergence and improved execution compared to traditional gradient descent algorithms.
- Adam optimizer regulates the learning rates based on past gradients, making it suitable for a wide range of problems and architectures.

**Loss function:**

- The loss function measures how the model’s performance is evaluated during training.
- In this case, the Mean Squared Error (MSE) loss function is used.
- MSE is frequently used for regression tasks, including sequence prediction, where the goal is to minimize the squared difference between predicted and true values.
- The model learns to make forecasts that are closer to the ground truth values by minimizing the MSE loss during training, thereby improving its accuracy over time.

## Results, discussion and conclusion

### Results and discussion

Following metrics have been used to measure the performance of models:

Proposed and existing models were evaluated using metrics mentioned in Table 1. The model was trained for 10 epochs using the “MSE” loss function and the “Adam” optimizer. During training, the loss was approximately 0.0003, while the average loss was 0.0008. Using the same data and the training epochs, the ANN (Artificial Neural Network) testing loss was 0.0011, while the RNN-attention and GRU-attention testing losses were 0.0008 and 0.0005, respectively. Table 2 shows the training, validation, and testing results of all four models usder study, as follows:

**Table 1:** List of metrics used for comparison of models.

Sr #	Metrics	Abbreviations
1	MAE	Mean Absolute Error
2	MAPE	Mean Absolute Percentage Error
3	MSE	Mean Squared Error
4	RMSE	Root Mean Squared Error
5	$R^{2}$	R- Squared

**Table 2.** Training and testing loss of LSTM-attention model, ANN model, RNN-attention model and GRU-attention model.

Models	R^2^ (R-Squared Values)		
	Training	Validation	Testing
LSTM-attention	0.9996	0.9980	0.9921
ANN	0.9992	0.9934	0.9860
RNN-attention	0.9994	0.9957	0.9384
GRU-attention	0.9995	0.9956	0.9587

**Fig 4:**
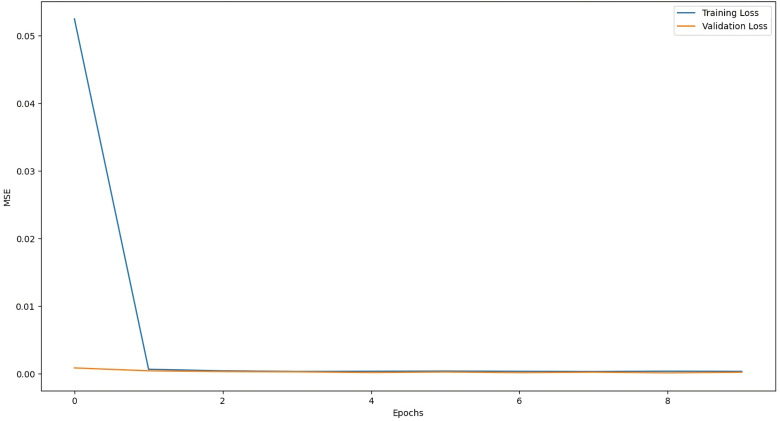
Decrease in loss during the training process.

**Fig 5:**
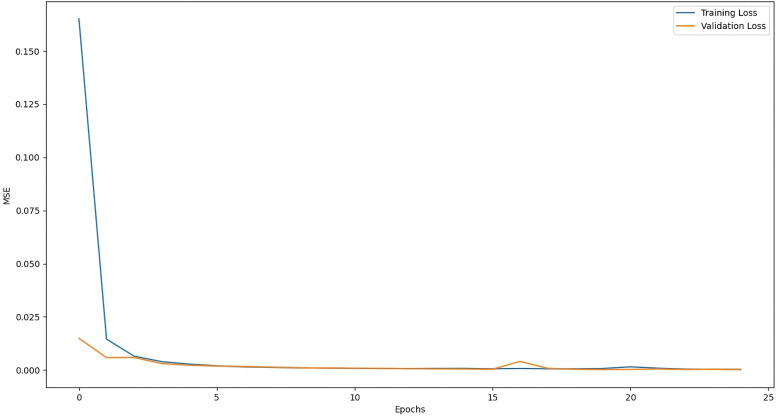
Training Loss of ANN model for 25 epochs.

**Fig 6:**
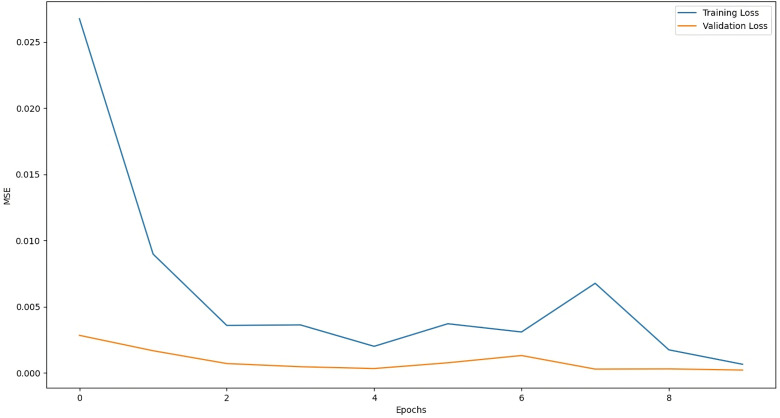
Training Loss of RNN-Attention.

**Fig 7:**
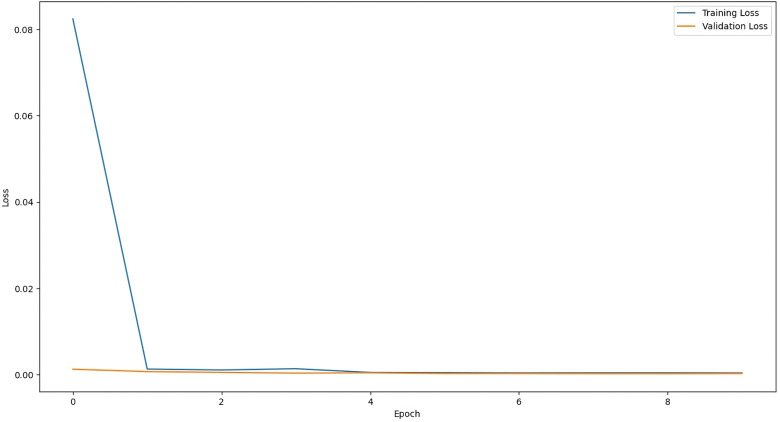
Training Loss of GRU-Attention.

The training graph of LSTM with Attention Model, ANN Model, RNN with Attention Model, and GRU with Attention Model are given below:

The above Fig 4 shows that the mean squared error function is decreasing during training, which means that the model is learning how to map input data to output data (prediction). In the early training cycle, the training is fast but gradually decreases, showing that the loss function is approaching the absolute minimum. Fig 5 shows the training loss of the ANN model along with training epochs. After training for a higher number of epochs (training cycles), the loss of the ANN model is very close to the absolute minimum. Similarly, Fig 6 and Fig 7 represent the training loss of RNN with Attention and GRU with Attention, respectively, for 10 epochs.

Table 3 represents the metrics of testing data generated by LSTM with Attention, the metrics of testing data generated by ANN, the metrics of testing data generated by RNN-attention, and the metrics of testing data generated by GRU-attention:

**Table 3.** Metrics of Testing Data generated by LSTM-Attention,ANN, RNN-attention and GRU-Attention. .

Models	Metrices				
	RMSE	MAE	MAPE	MSE	$R^{2}$
LSTM-attention	0.0245	0.0212	2.6491	0.0008	0.9921
ANN	0.0324	0.0246	2.6361	0.0011	0.9861
RNN-attention	0.0294	0.0229	2.1249	0.0008	0.9384
GRU-attention	0.0244	0.0187	1.7404	0.0005	0.9587

**Fig 8:**
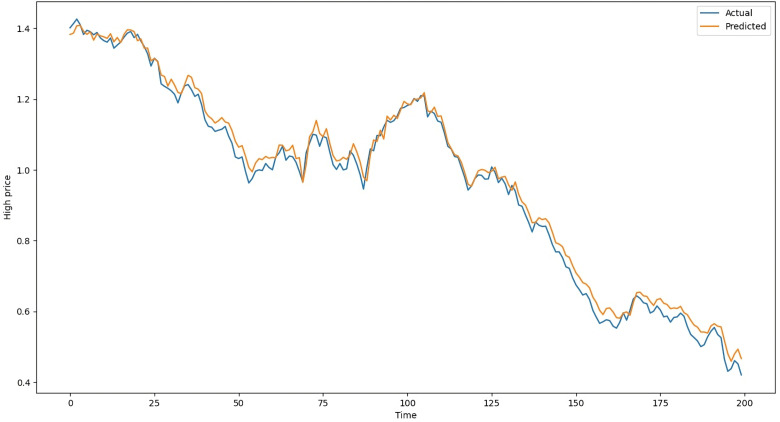
Actual and Predicted Values by the LSTM-Attention Model.

**Fig 9:**
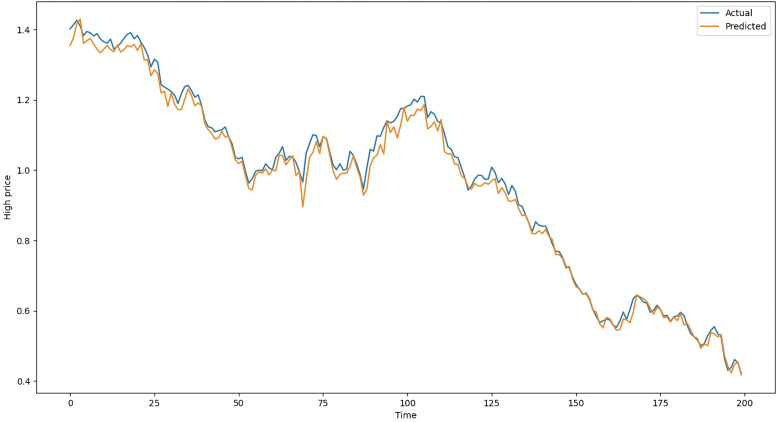
Actual and Predicted Values by the ANN Model.

**Fig 10:**
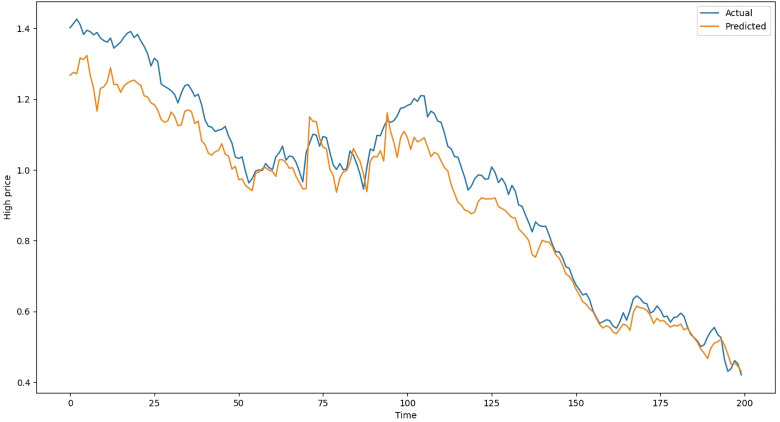
Actual and Predicted Values by the RNN-Attention Model.

**Fig 11:**
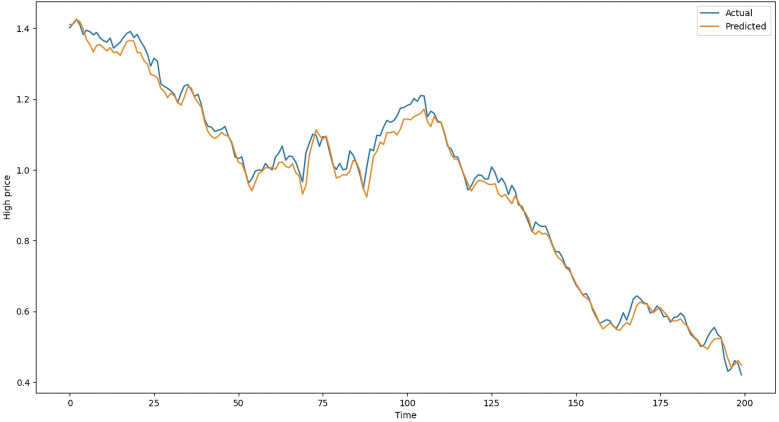
Actual and Predicted Values by the GRU-Attention Model.

The difference between estimated values and actual values in the testing data of LSTM with Attention on average is 104 points, whereas it is 109 points with ANN, 119 points with RNN-Attention, and 105 points with GRU-Attention. Table 3 shows the results of the models on the testing dataset. The above metrics are calculated by comparing the actual standardized testing data with the predicted data from the ANN, LSTM-Attention, RNN-Attention, and GRU-Attention models. Following Fig 8 is the chart of the actual and predicted values of LSTM with Attention, ranging from May 7, 2020, to February 23, 2021. Similarly, Fig 9 is the chart of the actual and predicted values of ANN, Fig 10 is the chart of the actual and predicted values of RNN with Attention, and Fig 11 is the graphical representation of the actual and predicted values of GRU with Attention, respectively, given below:

In Fig 8, the vertical axis shows the “High” column while the horizontal axis represents the time (Days). The blue line shows the actual “High” values and the orange line shows the predictions of the LSTM-Attention Model. Both of them are closed together which is also evident from Table 3. Figs 9–11 show the charts of ANN, RNN-Attention, and GRU-Attention, respectively, for predicting the same data:

Fig 9 shows that the ANN model’s predictions exhibit fluctuations (jumps), whereas the predictions of the LSTM-Attention model are smoother. As a result, the ANN model has a lower R^2^ and higher Mean Square, as presenting in Table 3. If the **ANN model is trained** for 25 epochs (training cycles), the **R**^**2**^ value improves to **0.9921**.

Figs 10 and 11 show the actual and predicted data of the RNN-Attention Model and GRU-Attention Model, respectively, which are less precise than those of the LSTM-Attention Model. An important point to note in Table 3 in the evaluation MSE metrics of GRU-Attention, which are lower than those of the LSTM-Attention model. However, for sequential time series data, GRU-Attention does not provide consistent results and outcomes may vary across different training sessions.

### SHAP value of proposed model architecture

**Fig 12:**
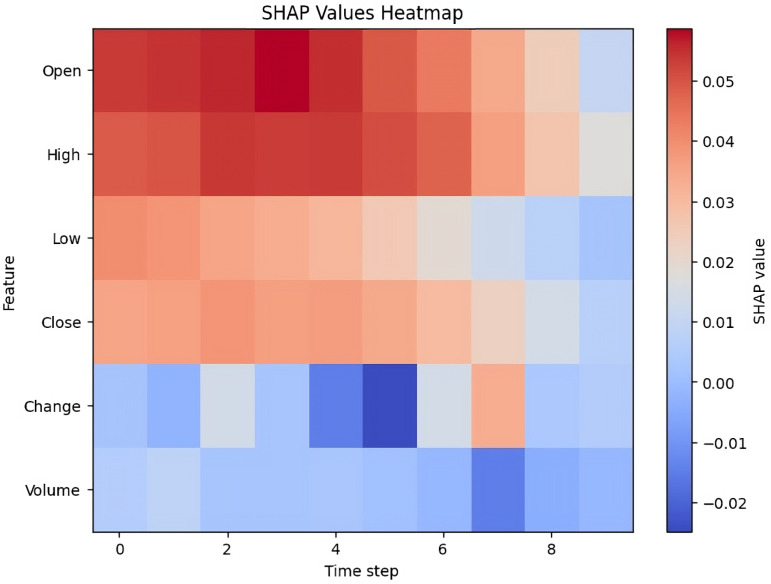
SHAP Value Heatmap.

**Fig 13:**
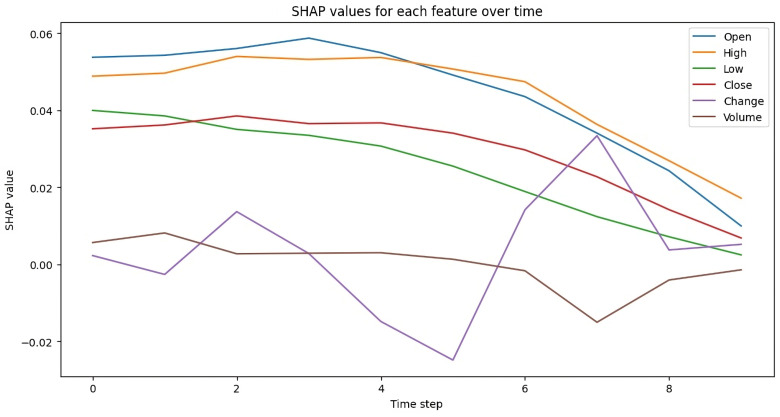
SHAP Value Line Graph.

Detailed SHAP value analysis is used to evaluate the performance of the proposed model and the contribution of associated features predicting stock prices. Fig 12 shows the heatmap of stock exchange features involvement in the model, and Fig 13 represents the detailed line graph analysis of the SHAP value as described below:

**Heatmap breakdown:**

**X-axis:**
Displays the time steps (0–9).
**Y-axis:**
Dataset’s features, including Open, High, Low, Close, Change, and Volume.

#### Color scale.

**Red (positive SHAP values)** indicates positive SHAP values, meaning the feature contributes positively to the model’s prediction.

**Blue (negative SHAP values)** indicates negative SHAP values, showing that the feature negatively impacts the model’s output.

**The intensity of the color** reflects the strength of the contribution, with deeper colors representing higher SHAP values

**Feature-wise analysis:**

1. **Open:**

This feature shows strong positive SHAP values, especially during the early time steps (0–6), indicating a significant positive role in the model’s predictions.

1. **High:**

Like the Open feature, it positively influences the model’s output across all time steps, though its impact diminishes slightly after step 6.

1. **Low:**

Although the SHAP values for Low are generally positive, they are weaker than those of Open and High. Its contribution is stronger at the beginning but gradually declines over time.

1. **Close:**

This feature moderately affects the predictions in a positive way. while less influential compared to Open and High, it remains steady throughout the time series.

1. **Change:**

This feature is the most unpredictable, with SHAP values fluctuating between positive and negative. Between steps 4 and 6, it shows a strong negative impact (dark blue), indicating a significant role in reducing the model’s output during that period. The model appears particularly sensitive to this feature at these steps.

1. **Volume**

The Volume feature generally has negative SHAP values, indicating a slight but consistent negative influence on the model’s predictions. Its overall effect is minimal compared to the other features, as reflected by the lighter color Intensity.

**General Observations:**

- **Open, High, and Close** consistently have a positive impact on the predictions, particularly during the initial time steps.
- **Change** shows the most variability, with both positive and negative effects depending on the time step, and it has a notable influence between steps 4 and 6.
- **Volume** has the smallest overall effect, with low SHAP values throughout the time series, indicating minimal contribution to the predictions.

The model places significant emphasis on price-related features (Open, High, Low, Close), particularly in the earlier time steps. It also shows greater sensitivity to changes during the mid to late stages of the series, especially regarding the Change feature. According to the heatmap, Volume contributes less significantly to the model’s predictions.

## Conclusion

The proposed neural network architecture, tailored for sequence prediction tasks using Karachi Stock Exchange data, demonstrates promising performance metrics, highlighting its ability to capture intricate dependencies in sequential data. By integrating LSTM units with an attention mechanism, the model effectively identifies temporal patterns and selectively focuses on critical features for accurate predictions. The LSTM-Attention model achieved strong performance, with a root mean squared error (RMSE) of 0.0245, mean absolute error (MAE) of 0.0212, mean absolute percentage error (MAPE) of 2.6491%, and mean squared error (MSE) of 0.0008. These metrics underline the effectiveness of leveraging attention mechanisms within the LSTM architecture, ensuring the model generalizes well to unseen data while delivering reliable forecasts in a dynamic stock market environment. Potential applications include predictive modeling, time series forecasting, and real-time financial monitoring systems. Future research could explore incorporating additional economic indicators, such as GDP, inflation, stock-related news, and political factors, as well as further improving the model’s attention mechanism.

## Abbreviations

KSE: Karachi Stock Exchange
PSX: Pakistan Stock Exchange
ANN: Artificial Neural Network
LSTM: Long Short-Term Memory
RNN: Recurrent Neural Network
GRU: Gated Recurrent Unit
MAE: Mean Absolute Average
MAPE: Mean Average Percentage Error
MSE: Mean Square Error
RMSE: Root Mean Squared Error

## References

[1] Usmani M, Adil SH, Raza K, Ali SSA. Stock market prediction using machine learning techniques. 2016 3rd International Conference on Computer and Information Sciences (ICCOINS). 2016:322–7.

[2] Rasel RI, Sultana N, Meesad PJ. An efficient modelling approach for forecasting financial time series data using support vector regression and windowing operators. J Comput Intell Syst. 2015;4(2):134–50.

[3] Kang Q, Chen EJ, Li Z-C, Luo H-B, U YJ, Liu S. Attention-based LSTM predictive model for the attitude and position of shield machine in tunneling. Underground Space. 2023;13:335–50.

[4] Zaffar A, Hussain SMA. Modeling and prediction of KSE - 100 index closing based on news sentiments: an applications of machine learning model and ARMA (p, q) model. Multimed Tools Appl. 2022;81(23):33311–33. doi: 10.1007/s11042-022-13052-2 35463220 PMC9013547

[5] Hasan N, Rasel RI. Artificial neural network approach for stock price and trend prediction. International Conference on Advanced Information & Communication Technology. 2016;5. doi: DOIoridentifierneeded

[6] Aslam M, Lee S-J, Khang S-H, Hong S. Two-Stage Attention Over LSTM With Bayesian Optimization for Day-Ahead Solar Power Forecasting. IEEE Access. 2021;9:107387–98. doi: 10.1109/access.2021.3100105

[7] Qin C, Shi G, Tao J, Yu H, Jin Y, Lei J, et al. Precise cutterhead torque prediction for shield tunneling machines using a novel hybrid deep neural network. Mechanical Systems and Signal Processing. 2021;151:107386. doi: 10.1016/j.ymssp.2020.107386

[8] Zhou L, Mao Q, Huang X, Zhang F, Zhang Z. Feature refinement: An expression-specific feature learning and fusion method for micro-expression recognition. Pattern Recognit. 2022;122:108275. doi: 10.1016/j.patcog.2021.108275

[9] Chen S, Ge LJQF. Exploring the attention mechanism in LSTM-based Hong Kong stock price movement prediction. Journal of Financial Markets. 2019;19(9):1507–15.

[10] Liu J-G, Yang Z-H, Li S-N, Yu C-R. A generative model for the collective attention of the Chinese stock market investors. Physica A: Stat Mech Appl. 2018;512:1175–82. doi: 10.1016/j.physa.2018.08.036

[11] Shi M, Yang B, Chen R, Ye D. Logging curve prediction method based on CNN-LSTM-attention. Earth Sci Inform. 2022;15(4):2119–31. doi: 10.1007/s12145-022-00864-x

[12] Xiang S, Qin Y, Zhu C, Wang Y, Chen H. Long short-term memory neural network with weight amplification and its application into gear remaining useful life prediction. Eng Appl Artif Intell. 2020;91:103587. doi: 10.1016/j.engappai.2020.103587

[13] Diao Y, et al. Emotion cause detection with enhanced-representation attention convolutional-context network. Soft Comput. 2021;25:1297–307.

[14] Atsalakis GS, Valavanis KP. Surveying stock market forecasting techniques–Part II: Soft computing methods. Journal of Computational and Financial Analysis. 2009;36(3):5932–41.

[15] Zhou X, Pan Z, Hu G, Tang S, Zhao CJMPIE. “Stock market prediction on high‐frequency data using generative adversarial nets”. 2018; 1:4907423.

[16] Rasool SA, Kiani AKJS. Stock returns prediction by using artificial neural network model for Pakistan stock exchange. J Manag Sci. 2018;4(2):190–201.

[17] Hochreiter S. J. N. C. M.-P. “Long Short-term Memory.” 1997. 10.1162/neco.1997.9.8.1735 9377276

[18] Vaswani AJAINIPS. Attention is all you need. Proceedings of the Conference on Neural Information Processing Systems. 2017;30:5998–6008.

[19] Selvin S, Vinayakumar R, Gopalakrishnan E, Menon VK, Soman K. Stock price prediction using LSTM, RNN and CNN-sliding window model. 2017 International Conference on Advances in Computing, Communications and Informatics (ICACCI). 2017;1643–7.

[20] Gers FA, Schmidhuber J, Cummins F. Learning to forget: continual prediction with LSTM. Neural Comput. 2000;12(10):2451–71. doi: 10.1162/089976600300015015 11032042

[21] Mahmoodzadeh A, Nejati HR, Mohammadi M, Ibrahim HH, Rashidi S, Rashid TW. Forecasting tunnel boring machine penetration rate using LSTM deep neural network optimized by grey wolf optimization algorithm. J Constr Eng Manag. 2022;209:118303.

[22] Ordóñez FJ, Roggen D. Deep Convolutional and LSTM Recurrent Neural Networks for Multimodal Wearable Activity Recognition. Sensors (Basel). 2016;16(1):115. doi: 10.3390/s16010115 26797612 PMC4732148

[23] Shan F, He X, Armaghani DJ, Zhang P, Sheng DJT, Technology US. Success and challenges in predicting TBM penetration rate using recurrent neural networks. J Technol. 2022;130:104728.

[24] Bahdanau DJ a. p. a. Neural machine translation by jointly learning to align and translate.

[25] Liu Z, Zhou W, Li HJAT o. MC. AB-LSTM: Attention-based bidirectional LSTM model for scene text detection. Commun Appl. 2019;15(4):1–23.

[26] Luo T, Cao X, Li J, Dong K, Zhang R, Wei XJIDA. Multi-task prediction model based on ConvLSTM and encoder-decoder," 2021;25(2):359–382.

[27] Adhikari R, Agrawal RKJ a. p. a. An introductory study on time series modeling and forecasting. 2013.

[28] Meesad P, Rasel RI. Dhaka stock exchange trend analysis using support vector regression," In The 9th International Conference on Computing and InformationTechnology (IC2IT2013) 9th-10th May 2013 King Mongkut's University of Technology North Bangkok, Springer. 2013, pp. 135–143.

[29] Meesad P, Rasel RI. Predicting stock market price using support vector regression. 2013 International Conference on Informatics, Electronics and Vision (ICIEV), IEEE. 2013:1–6.

[30] Pai P-F, Lin C-S. A hybrid ARIMA and support vector machines model in stock price forecasting. Omega. 2005;33(6):497–505. doi: 10.1016/j.omega.2004.07.024

